# The mitochondrial genome of an Endangered freshwater snail *Koreoleptoxis nodifila* (Caenogastropoda: Semisulcospiridae) from South Korea

**DOI:** 10.1080/23802359.2021.1901626

**Published:** 2021-03-19

**Authors:** Eun Hwa Choi, Na Rae Choi, Ui Wook Hwang

**Affiliations:** a Department of Biology Education, Teachers College & Institute for Phylogenomics and Evolution, Kyungpook National University, Daegu, South Korea; b Institute for Korean Herb-Bio Convergence Promotion, Kyungpook National University, Daegu, South Korea; c Biomedical Convergence Science and Technology, Kyungpook National University, Daegu, South Korea

**Keywords:** *Koreoleptoxis nodifila*, Semisulcospiridae, Caeonogastropoda, mitochondrial genome, phylogeny

## Abstract

The mitochondrial genome of the Endangered freshwater snail *Koreoleptoxis nodifila* (Caenogastropoda: Semisulcospiridae) from South Korea is determined and characterized in detail. It is 15,737 bp in length being composed of 13 protein-coding genes (PCGs), 22 transfer *RNA* genes (tRNAs), two ribosomal *RNA* genes (rRNAs), and one control region. It has a base composition of 31.23% for A, 16.29% for G, 17.84% for C, and 34.64% for T. The phylogenetic trees reconstructed based on the maximum-likelihood (ML) method and Bayesian inference (BI) confirmed that *K. nodifila* belongs to the Semisulcospiridae clade in the monophyletic caeonogastropod superfamily Cerithioidea.

The family Semisulcospiridae, a member of Cerithioidea, is speciose and widely distributed throughout eastern Asia, including the Korean Peninsula, the Russian Far East, Japan, southern China, and Taiwan (Davis 1969; Chiu et al. 2017) as well as western North America (Strong and Köhler 2009). In South Korea, a total of seven species from the three semisulcospirid genera *Koreanomelania*, *Koreoleptoxis,* and *Semisulcospira* have been recorded so far (Ko et al. 2001; Lee et al. 2001). Recently, the genus *Koreanomelania* Burch & Jung, 1988 has been synonymized with the genus *Koreoleptoxis* Burch & Jung, 1988 based on the analyses of morphological data and molecular data, such as mitochondrial *COI* and *16S rRNA* (Köhler 2017).

During the last decades, mitochondrial genomes have been considered useful phylogenetic markers in mollusks (Lee et al. 2012; Zeng et al. 2015; Hilgers et al. 2016; Cha et al. 2018; Kim and Lee 2018; Jiang et al. 2019; Lee et al. 2019; Tenorio et al. 2020). Here, we characterized a nearly complete mitochondrial genome of the Endangered freshwater snail *Koreoleptoxis nodifila* (Martens 1886). The sample used for this study was collected from Yongtan-ri, Jeongseon-eup, Jeongseon-gun, Gangwon-do, South Korea (37°23′35.3′′N, 128°36′00.4′′E). The specimen was deposited under the voucher number FNRVIV0000000082 in the National Institute of Biological Resources (NIBR), Ministry of Environment, South Korea. Genomic DNA was extracted from the muscle tissue using a DNeasy Blood & Tissue kit (Qiagen, Hilden, Germany). Sequencing was performed from an Illumina paired-end library and 150 bp paired-end reads were generated through the Illumina NextSeq500 platform, resulting in a total of 81,273,381 reads (not published). For data assembly, sequencing errors were discarded using the error correction module of SOAPec version 2.02 (Luo et al. 2012). And then, mitochondrial genome assembly of *K. nodifila* was conducted using SOAPdenovo2 version 2.04-r240 (Luo et al. 2012). Reconstruction of the mitochondrial genome was performed with MITObim (Hahn et al. 2013) using the Illumina reads. The seed sequences were extended by mapping reads iteratively using MITObim. Finally, after conducting manual curation, the annotation of mitochondrial genes, such as protein-coding genes (PCGs), *tRNA* genes, and *rRNA* genes was carried out using the MITOS web server (Bernt et al. 2013). The mitogenome of *K. globus* (GenBank accession no. LC006055) was used as a reference for the annotation.

The mitochondrial genome of *K. nodifila* (GenBank accession no. NC_046494), which is 15,737 bp in length, exhibits the same gene components and gene order as those previously known from the family Semisulcospiridae, such as *Semisulcospira coreana* (Martens 1886) (Kim and Lee 2018), *S. libertina* (Zeng et al. 2015), and *Koreoleptoxis globus* (Martens 1886) (GenBank accession number LC006055; unpublished data). It contains a total of 37 genes including 13 PCGs (*COX1-3*, *ND1-6*, *ND4L*, *CYTB*, *ATP6*, and *ATP8*), two rRNAs (*16S rRNA* and *12S rRNA*), 22 tRNAs, and one control region, of which 16 genes are located on the positive strand and the remaining on the negative strand. The overall A + T content of the *K. nodifila* mitogenome is 65.87%: 64.66% for PCGs and 65.85% for tRNAs and rRNAs. The control region is located between *trnF* and *trnC.* Its size was predicted to be about 3 kb, out of which only 399 bp were sequenced. The control region sequencing could not be completed due to the A + T − rich hairpin structures and too long A + T − dinucleotide repeats. This is also observed in the semisulcospirid *K. globus* and the pachychilid *Tylomelania sarasinorum* (Hilgers et al. 2016).

The maximum likelihood (ML) and Bayesian inference (BI) trees were reconstructed based on the amino acid sequences of the 13 PCGs from 42 caenogastropod species (Figure 1). The best-fitting model mtZOA + F + I + G4 for the ML analysis was selected using the implemented function in IQ-Tree ( Trifinopoulos et al. 2016). We also performed BI analysis using MrBayes version 3.2.7a (Ronquist et al. 2012) under the best model mtREV + I + G4, each with four MCMC chains for 1,000,000 generations. Sampling was done at the end of every 1000th generation with 25% of the initial trees discarded as burnin. Both ML and BI trees strongly supported the monophyly of the subclass Caenogastropoda (100% BP − bootstrapping values in percent and 1.0 BPP – Bayesian posterior probability). It also confirmed that *K. nodifila* belongs to the Semisulcospiridae (100% BP and 1.0 BPP) in the monophyletic superfamily Cerithioidea (100% BP and 1.0 BPP) which includes Semisulcospiridae (*Semisulcospira* and *Koreoleptoxis*), Pachychilidae (*Tylomelania*), and Turritellidae (*Turritella*). Hypsogastropoda appeared as a monophyletic group (100% BP and 1.0 BPP), with exception of the superfamily Vermetoidea which placed at the basal branch of Caenogastropoda. Due to the unexpected placement of the superfamily Vermetoidea, the monophyly of the order Littorinimorpha was not supported, but was polyphyletic instead. The peculiar positioning of Vermetoidea, which is not in line with morphological evidence (Sigwart and Lindberg 2015), may be interpreted as a spurious result caused by the long-branch attraction artifact or some unique mitochondrial genome characteristics (i.e. Jiang et al. 2019). Within Hypsogastropoda excepting Vermetoidea, phylogenetic analyses did not recover Littorinimorpha as a monophyletic group, but rather suggested that this group is paraphyletic with respect to the order Neogastropoda. Whether the Littorinimorpha in this sense really is not a monophyletic groups needs further investigation. In addition, Architaenioglossa was not recovered as a monophyletic group because Cochlostomatidae was not placed in this clade. Compared to other studies addressing caenogastropod phylogeny based on mitochondrial genomes (Osca et al. 2015; Lee et al. 2019), the results presented here may be considered better-resolved because the monophyly of Viviparidae and Ampullariidae was recovered, although with somewhat low support in ML only (67% BP). Unexpectedly, within the order Neogastropoda, the monophylies of Conoidea and Volutoidea were not supported in the ML and BI analyses. These also need to be examined in detail in future studies.

**Figure 1.**
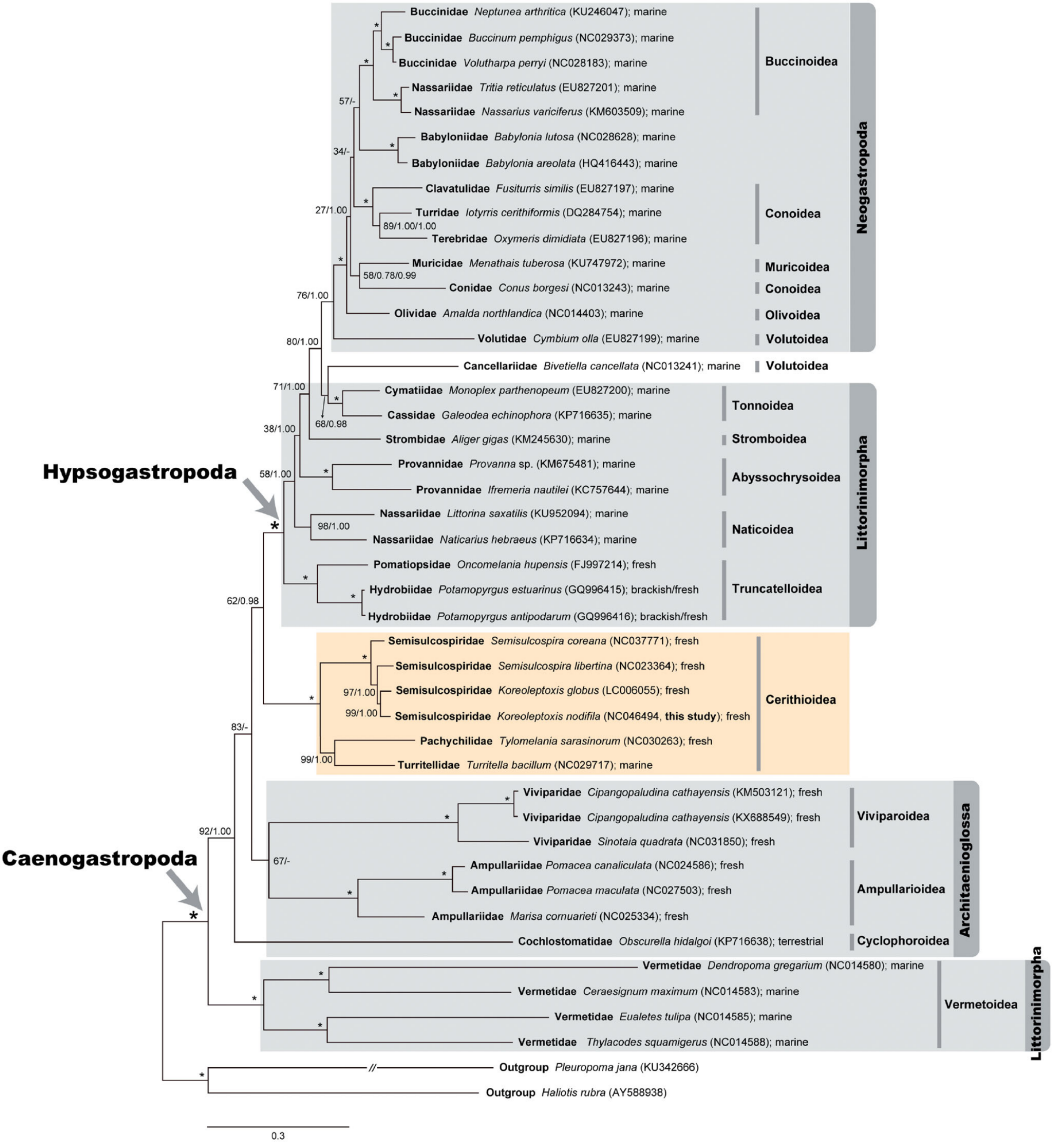
A maximum-likelihood tree reconstructed with the amino acid sequences of 13 mitochondrial PCGs showing relationships among 42 caenogastropod species. *Pleuropoma jana* (Neritimorpha) and *Haliotis rubra* (Vetigastropoda) are used as outgroups. The monophylies of Caenogastropoda and Hypsogastropoda are indicated with an arrow, respectively. The dark yellow box indicates the monophyly of the superfamily Cerithioidea including this result of *Koreoleptoxis nodifila*. Branch supports are bootstrapping values in percent (BP) obtained the ultrafast bootstrap method using IQ-TREE webserver and Bayesian posterior probability (BPP) inferred from Bayesian inference using MrBayes version 3.2.7a in order. The nodes exhibiting both 100 BP and 1.00 BPP mark an asterisk (*). Major habitats of the taxa examined here are depicted beside each taxon name.

## Data Availability

The data that support the findings of this study are openly available in GenBank of NCBI at https://www.ncbi.nlm.nih.gov/nuccore/NC_046494. The associated BioProject, SRA, and BioSample numbers are PRJNA693879, SRR13516403, and SAMN17487625, respectively.
